# Assessment of a novel deep learning-based software developed for automatic feature extraction and grading of radiographic knee osteoarthritis

**DOI:** 10.1186/s12891-023-06951-4

**Published:** 2023-11-08

**Authors:** Ji Soo Yoon, Chang-Jin Yon, Daewoo Lee, Jae Joon Lee, Chang Ho Kang, Seung-Baik Kang, Na-Kyoung Lee, Chong Bum Chang

**Affiliations:** 1 Department of Orthopaedic Surgery, Seoul National University Bundang Hospital, Seongnam-Si, Republic of Korea; 2 Department of Orthopaedic Surgery, Keimyung University Dongsan Hospital, Daegu, Republic of Korea; 3 Crescom, Seongnam-Si, Republic of Korea; 4 Department of Radiology, Korea University Anam Hospital, Seoul, Republic of Korea; 5 Department of Orthopaedic Surgery, SMG-SNU Boramae Medical Center, Seoul, Republic of Korea; 6 Department of Orthopaedic Surgery, Seoul National University College of Medicine, Seoul, Republic of Korea

**Keywords:** Deep learning, Artificial intelligence, Knee osteoarthritis, Joint space narrowing, Kellgren & Lawrence classification, KL grade

## Abstract

**Background:**

The Kellgren-Lawrence (KL) grading system is the most widely used method to classify the severity of osteoarthritis (OA) of the knee. However, due to ambiguity of terminology, the KL system showed inferior inter- and intra-observer reliability. For a more reliable evaluation, we recently developed novel deep learning (DL) software known as MediAI-OA to extract each radiographic feature of knee OA and to grade OA severity based on the KL system.

**Methods:**

This research used data from the Osteoarthritis Initiative for training and validation of MediAI-OA. 44,193 radiographs and 810 radiographs were set as the training data and used as validation data, respectively. This AI model was developed to automatically quantify the degree of joint space narrowing (JSN) of medial and lateral tibiofemoral joint, to automatically detect osteophytes in four regions (medial distal femur, lateral distal femur, medial proximal tibia and lateral proximal tibia) of the knee joint, to classify the KL grade, and present the results of these three OA features together. The model was tested by using 400 test datasets, and the results were compared to the ground truth. The accuracy of the JSN quantification and osteophyte detection was evaluated. The KL grade classification performance was evaluated by precision, recall, F1 score, accuracy, and Cohen's kappa coefficient. In addition, we defined KL grade 2 or higher as clinically significant OA, and accuracy of OA diagnosis were obtained.

**Results:**

The mean squared error of JSN rate quantification was 0.067 and average osteophyte detection accuracy of the MediAI-OA was 0.84. The accuracy of KL grading was 0.83, and the kappa coefficient between the AI model and ground truth was 0.768, which demonstrated substantial consistency. The OA diagnosis accuracy of this software was 0.92.

**Conclusions:**

The novel DL software known as MediAI-OA demonstrated satisfactory performance comparable to that of experienced orthopedic surgeons and radiologists for analyzing features of knee OA, KL grading and OA diagnosis. Therefore, reliable KL grading can be performed and the burden of the radiologist can be reduced by using MediAI-OA.

## Background

Osteoarthritis (OA) of the knee is highly prevalent and can lead to severe pain and significant functional impairments in patients [1]. Coupled with increasing life expectancy, the prevalence of knee OA is also increasing and has become one of the most important diseases that causes disability in middle-aged and elderly people, which correspondingly leads to considerable social economic costs [2–5].

The most important and practical method for the diagnosis of knee OA is the use of plane knee radiographs. Among the instruments for grading radiographic knee OA, the Kellgren-Lawrence (KL) grading system is the most commonly used method because it is simple and practical [6, 7]. However, there are some limitations in grading knee OA with this system. For example, KL system classifies grades based on two important features of OA, the degree of joint space narrowing (JSN) and the degree of osteophyte formation, but each grading is defined with ambiguous terms, such as “possible”, “doubtful”, “definite”, “moderate” and “severe” [6]. Therefore, the KL system has shown inferior interobserver and intraobserver reliability [8–10].

For a more reliable evaluation of OA grading with the KL system, there has been an effort to develop an automated system by using deep learning (DL) software, and recent studies have demonstrated promising results in grading OA [11–16]. However, most of the KL grading DL software reported thus far presents only the KL grading results [11, 13–16]. When taking plain radiographs serially over time in a patient, it was thought that quantitative measurements of OA features such as the degree of JSN or osteophytes would be necessary to determine whether the OA is progressing further within the same grade. In addition, in Korea's National Health Insurance, the K-L grade criteria for performing total knee arthroplasty, unicompartmental knee arthroplasty, or high tibial osteotomy vary depending on the patient's age. Therefore, it is important to determine K-L grade more accurately by quantifying or semi-quantifying key features. Therefore, we have developed novel DL software known as MediAI-OA to assess radiographic features of knee OA, including osteophyte and joint space narrowing, as well as grading of OA severity, based on the KL system. In this study, we aimed to introduce this novel DL software for the first time and to evaluate the accuracy and performance of this model for KL grading of knee OA.

## Methods

### Ethical approval

This study was approved by the Institutional Review Board of Seoul National University Bundang Hospital. The requirement for informed consent was waived because of the retrospective nature of the study (Institutional Review Board Number: B-2106–691-104).

### Dataset

This research used data from the Osteoarthritis Initiative (OAI) for training and validation of the MediAI-OA. The OAI (https://nda.nih.gov/oai/) is a multicenter, longitudinal, prospective observational study of knee OA [17]. Radiographic features from weight-bearing fixed flexion knee posteroanterior (PA) radiographs, such as osteophytes, attrition, joint space width and KL grade, were provided in this dataset. Among the OAI datasets, 44,193 out of 45,003 radiographs were set as the training data, and 810 radiographs were used as validation data. These data include all KL grades 0 to 4, and training set and validation set were randomly extracted from each grade. The purpose of using the validation dataset in the process of developing this model was to determine the optimal weight value and parameters. In addition, down-sampling was performed to improve data imbalance by equalizing the number of validation samples for each KL grade to prevent the model from being biased towards a specific KL grade.

To construct a dataset for testing this novel DL software, patients aged 20 years and older who visited our institution for knee pain from 2019 to 2021 were retrospectively reviewed. Among these patients, only those patients with standing knee anteroposterior (AP) radiographs and Rosenberg (45-degree flexion standing PA view) radiographs were selected. The following patients were excluded from the analysis: 1) patients diagnosed with inflammatory arthritis; 2) patients who underwent knee arthroplasty; 3) patients with internal metal fixation devices around the knee due to previous surgery; 4) patients who had bony deformities due to previous fractures, tumors or infections around the knee; and 5) patients with only a radiograph, which can give a large error in the KL grading classification due to the severely inappropriate angle of the X-ray beam during imaging. The same exclusion criteria were applied to the training dataset and validation dataset. Finally, a total of 400 knee radiographs (200 standing AP and 200 Rosenberg radiographs) from 102 patients were selected as the test set.

### Software and libraries

The following software and libraries were used during the MediAI-OA development process: Keras 2.3.1, Tensorflow 2.0.0, Scikit-learn 0.24.2, Torch 1.7.0, Torchvision 0.8.1, Numpy 1.19.5, and Opencv 4.5.5.64.

### Ground truth

In the test set radiographs, minimum joint space width (mJSW) measurements, the determination of osteophyte presence and final KL grading were performed by two orthopedic knee arthroplasty specialist (CBC and SBK) and one radiologist of musculoskeletal department (CHK) with more than 20 years of experience. The mJSW was determined as the narrowest knee joint space between medial tibiofemoral joint or lateral tibiofemoral joint. To quantify the mJSW, one radiologist (CHK) directly segmented the joint space, and the software performed the quantification. The presence of osteophytes was separately assessed in the following four regions: medial distal femur (MF), lateral distal femur (LF), medial proximal tibia (MT) and lateral proximal tibia (LT). KL grading was performed according to the original KL system [6, 18]: grade 0 indicated a definite absence of evidence of osteoarthritis; grade 1 demonstrated doubtful narrowing of the joint space and possible osteophyte lipping; grade 2 demonstrated definite osteophytes and possible (< 50%) JSN; grade 3 was defined as moderate multiple osteophytes, definite (> 50%) JSN, some sclerosis and possible deformity of bone ends; and grade 4 was defined as large osteophytes, marked JSN in which there was bone-to-bone contact of the tibiofemoral joint [19], severe sclerosis and definite deformity of the bone ends. Before the evaluation of the entire test set, a pilot test was conducted with a part of the test set, and consensus among three evaluators was established. The result that was agreed upon by two or more evaluators was adopted as the ground truth; in addition, if there was a disagreement between all three evaluators, re-evaluation was performed. If the discrepancy persisted, grade was determined via consensus meeting.

### Data preprocessing & augmentations

Data preprocessing and data augmentation were performed to improve the learning performance of the MediAI-OA. First, when both knees were taken together in a plain radiograph, one side was separated and processed. Afterwards, the left knee image was inverted to be in the same direction as the right knee image. Zero padding was applied to the images to retain in-plain dimensions [20]. In addition, normalization, adaptive histogram equalization, brightness change, contrast change and noise addition were applied to data via online augmentation [21]. During the model test, augmentation was applied to the test dataset, and the results were derived by ensemble them.

### The algorithm for knee osteoarthritis severity analysis

**Fig. 1:**
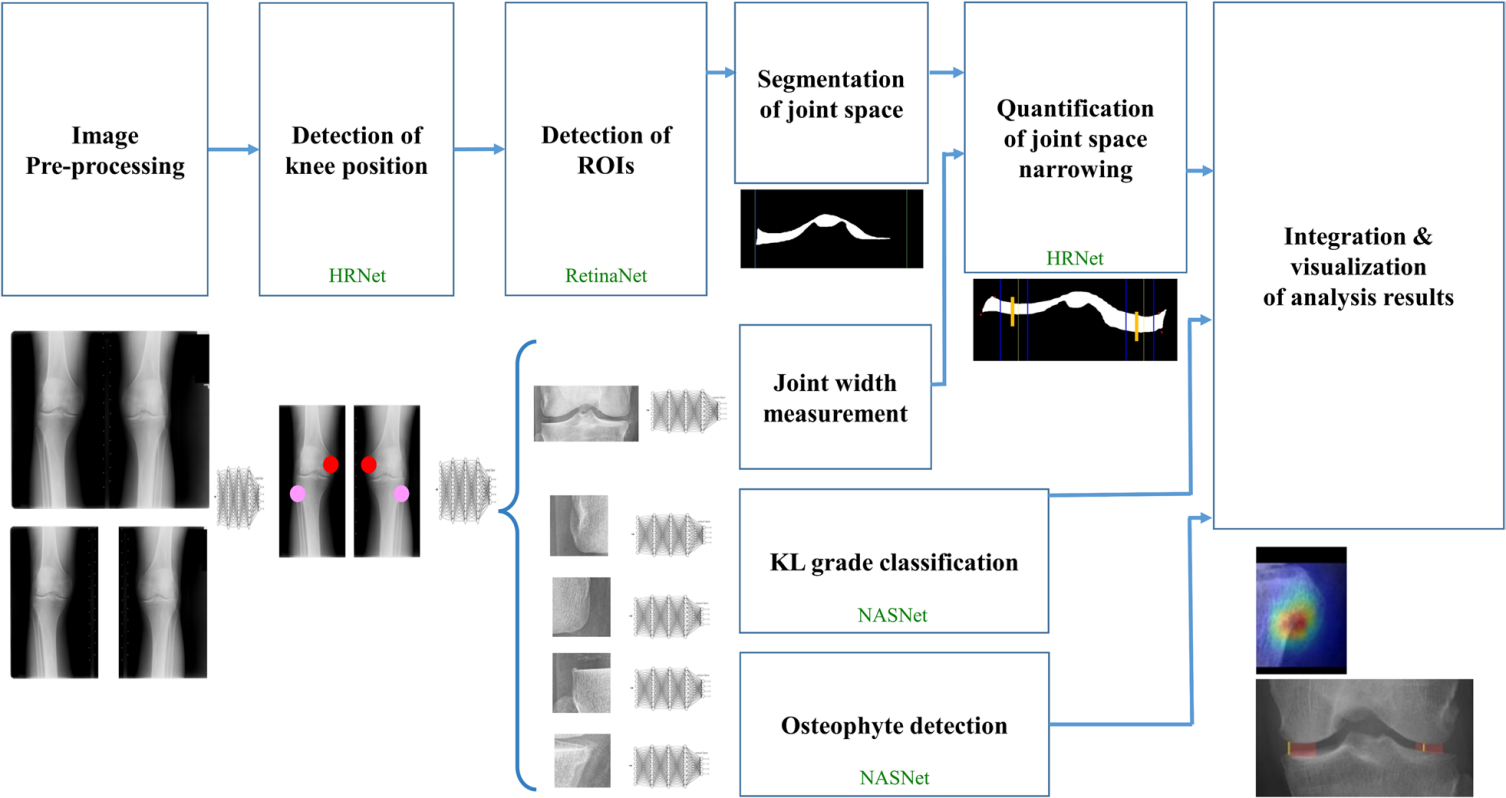
Schematic diagram of the novel DL software known as MediAI-OA. This model preprocesses knee radiographs and detects knee positions and ROIs. Subsequently, the quantification of the joint space narrowing, detection of osteophytes and classification of KL grade are automatically performed. The results are visualized in an integrated manner. The figure shows which processes the existing network architecture model was used for. HRNet was used to detect knee position and automatically measure tibia width for quantification of joint space narrowing. RetinaNet was used for detection of ROIs and NASNet was used for KL grade classification and osteophyte detection

The MediAI-OA approach consists of the following 4 steps: (1) detection of the knee joint region and regions associated with osteophytes; (2) segmentation and quantitative analysis of the joint space width area; (3) determination of the presence of osteophytes in each region (MF, LF, MT and LT); (4) automatic classification of KL grade; and (5) integration and visualization of OA features of the knee joint. Figure 1 shows the entire pipeline. We used high-resolution network (HRNet), RetinaNet, and Neural Architecture Search Network (NASNet) models in the development of each process, and these models are all pretrained models using the OAI dataset. For the NasNet model, KL grade and presence of osteophytes were used as given in the OAI dataset. For HRNet and RetinaNet models for detection of regions of interest (ROIs), the authors manually annotated the region and the model was trained with these data.

### Step 1: Detection of knee position and ROIs

In order to determine whether the radiograph is a right knee image, a left knee image, or an image that includes both knees, the model was trained to detect medial femoral condyle and center of the fibular head of each knee by using a key point detection model. A HRNet was used for key point detection, which has the advantage of using less memory while maintaining high performance [22].

**Fig. 2:**
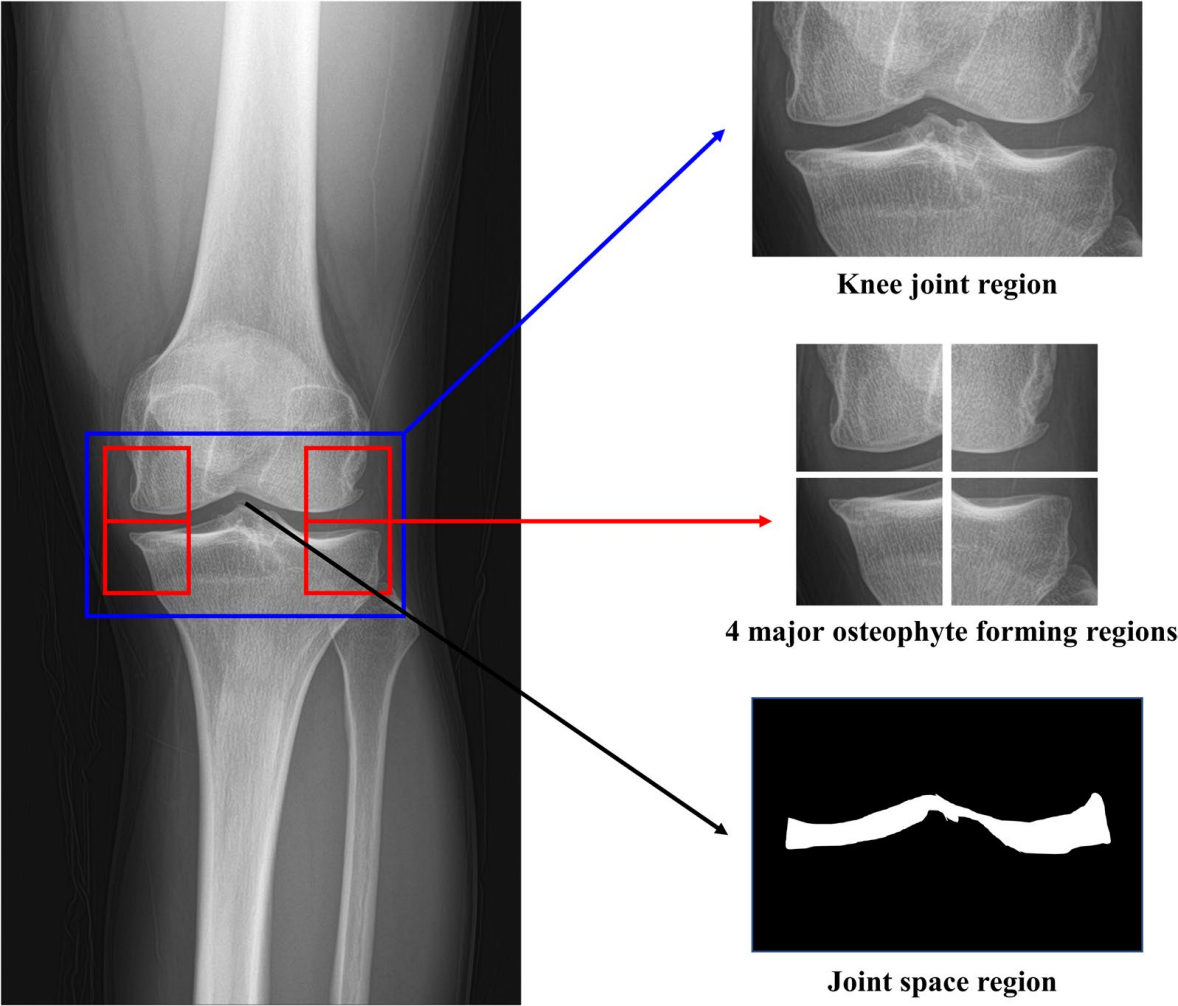
Five regions of interests (ROIs). The knee joint space region and four major osteophyte regions, including medial distal femur, lateral distal femur, medial proximal tibia and lateral proximal tibia, were set as the ROIs

We assigned ROIs to the training data image through bounding box annotation and trained MediAI-OA to automatically detect ROIs by using RetinaNet [23]. RetinaNet showed better performance in terms of speed and accuracy than Faster R-CNN when applied during the development process, therefore, RetinaNet was used. The following five areas were trained ROIs: an area that includes the distal femoral condyle and the proximal tibial plateau centered on the knee joint space and the four major osteophyte sites, including the MF, LF, MT and LT (Fig. 2). These four osteophyte ROIs are key areas for determining the grade of arthritis radiologically.

### Step 2: Automatic segmentation of joint space & quantification

**Fig. 3:**
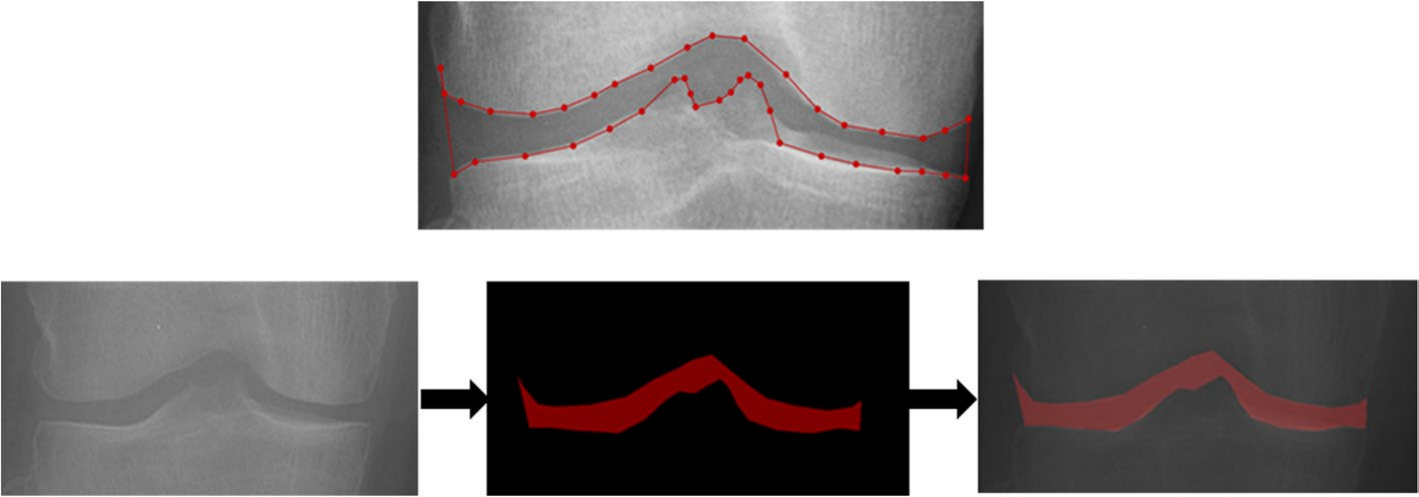
The automatic segmentation process of the knee joint space

**Fig. 4:**
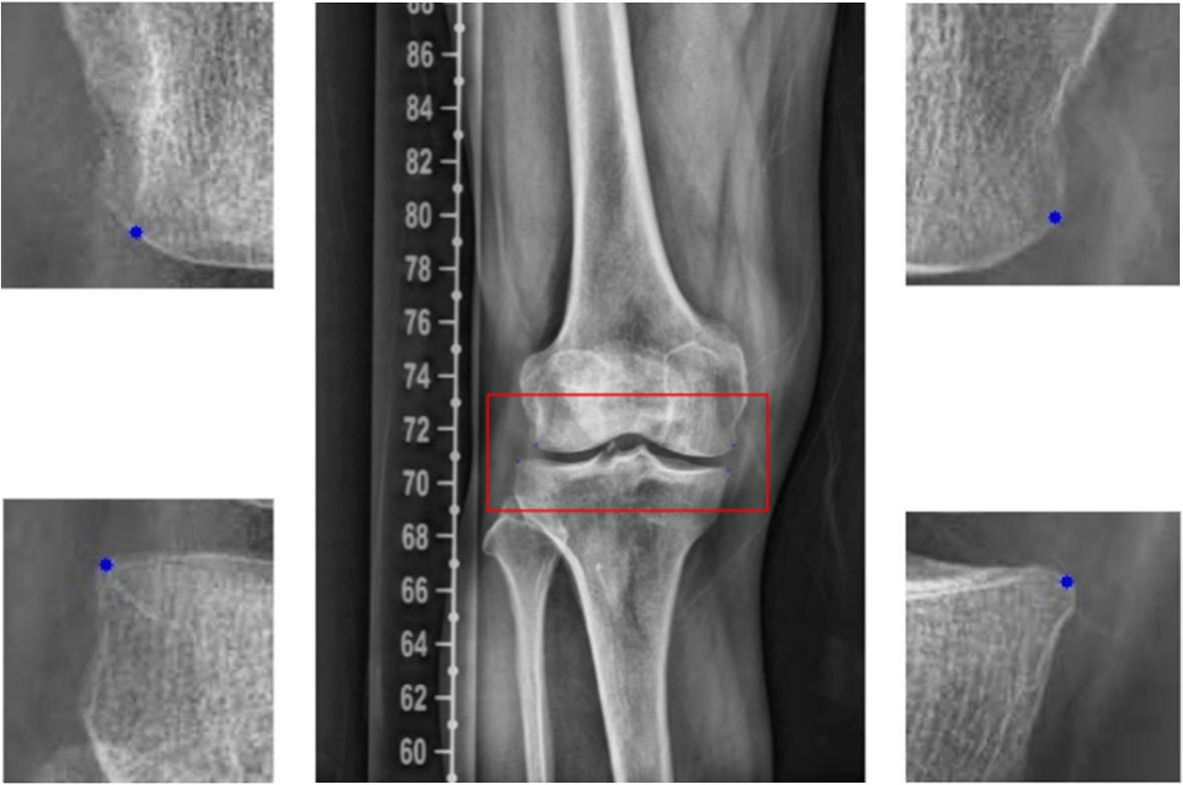
The result of four corner detection in the knee. To determine the boundary of the joint area and to measure the tibial plateau width, the AI model was trained to detect four corners. The length of the line connecting two corners of the tibia was used as the tibial width

**Fig. 5:**
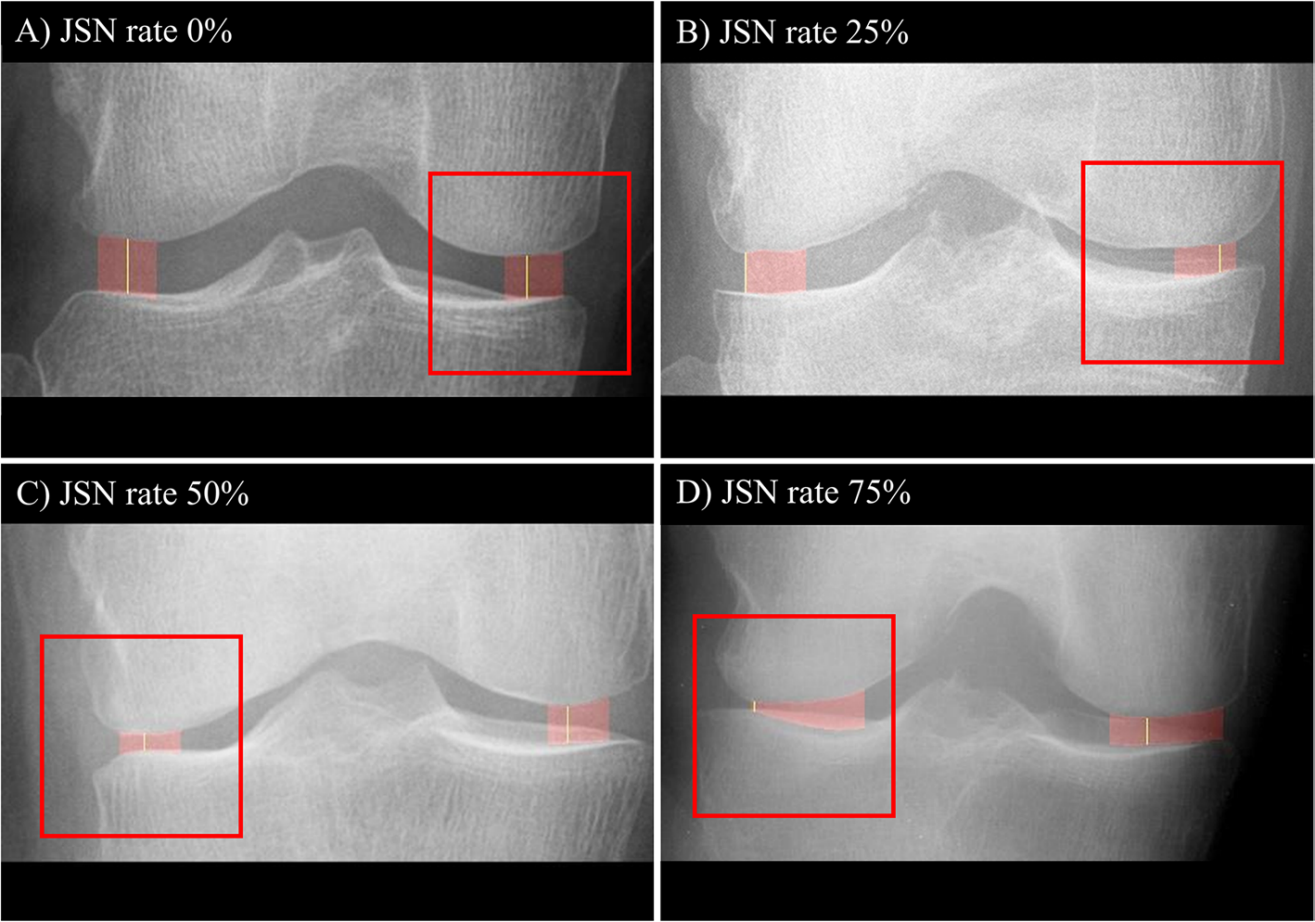
Example radiographs with JSN rates **A**) 0%, **B**) 25%, **C**) 50%, and **D**) 75%. Among medial and lateral joint space, the red square box is the area corresponding to each percentage

This AI model was developed to automatically segment the knee joint space region for the quantification of mJSW (Fig. 3). In the segmented joint space area, both the narrowest medial joint space width and lateral joint space width were calculated in the peripheral part where the femoral condyle and tibial plateau form a joint, except for the central part where the femoral notch and tibial spine exist. In this study, mJSW was measured as the ratio of the narrowest medial or lateral tibiofemoral joint space width to the mediolateral width of the tibial plateau to correct for possible individual variations in joint space width [24]. The AI model was also trained to detect most medial and most lateral corner points of the distal femur and proximal tibia to determine the boundary of the joint area by using HRNet (as shown in Fig. 4). In addition, the tibial plateau width was also measured through the points detected in this process. The length of the line connecting the bottom 2 corners among the 4 corners was used as the tibial width. Subsequently, to quantify the degree of JSN, JSN rate was calculated by comparing it with the mJSW ratio of the normal population. The mJSW of the normal population was defined as the median value of mJSW in the KL grade 0 dataset. The JSN rate was defined as 0% when there was no JSN compared with normal population, and when complete bone-to-bone contact between femur and tibia occurred, the JSN rate was defined as 100% (Fig. 5).

$${\varvec{J}}{\varvec{S}}{\varvec{N}}\boldsymbol{ }{\varvec{r}}{\varvec{a}}{\varvec{t}}{\varvec{e}}\boldsymbol{ }\left({\varvec{x}}\right)=100\times \left(1-\frac{{{\varvec{m}}{\varvec{J}}{\varvec{S}}{\varvec{W}}}_{{\varvec{x}}}}{{{\varvec{m}}{\varvec{e}}{\varvec{d}}{\varvec{i}}{\varvec{a}}{\varvec{n}}\boldsymbol{ }{\varvec{v}}{\varvec{a}}{\varvec{l}}{\varvec{u}}{\varvec{e}}\boldsymbol{ }{\varvec{o}}{\varvec{f}}\boldsymbol{ }{\varvec{m}}{\varvec{J}}{\varvec{S}}{\varvec{W}}}_{{\varvec{K}}{\varvec{L}}0}}\right)$$

$$=100\boldsymbol{ }\times (1-\frac{{{\varvec{n}}{\varvec{a}}{\varvec{r}}{\varvec{r}}{\varvec{o}}{\varvec{w}}{\varvec{e}}{\varvec{s}}{\varvec{t}}\boldsymbol{ }{\varvec{J}}{\varvec{S}}{\varvec{W}}}_{{\varvec{x}}}/{{\varvec{t}}{\varvec{i}}{\varvec{b}}{\varvec{i}}{\varvec{a}}{\varvec{l}}\boldsymbol{ }{\varvec{w}}{\varvec{i}}{\varvec{d}}{\varvec{t}}{\varvec{h}}}_{{\varvec{x}}}}{{{\varvec{M}}{\varvec{e}}{\varvec{d}}{\varvec{i}}{\varvec{a}}{\varvec{n}}\boldsymbol{ }{\varvec{v}}{\varvec{a}}{\varvec{l}}{\varvec{u}}{\varvec{e}}\boldsymbol{ }[({\varvec{n}}{\varvec{a}}{\varvec{r}}{\varvec{r}}{\varvec{o}}{\varvec{w}}{\varvec{e}}{\varvec{s}}{\varvec{t}}\boldsymbol{ }{\varvec{J}}{\varvec{S}}{\varvec{W}}/{\varvec{t}}{\varvec{i}}{\varvec{b}}{\varvec{i}}{\varvec{a}}{\varvec{l}}\boldsymbol{ }{\varvec{w}}{\varvec{i}}{\varvec{d}}{\varvec{t}}{\varvec{h}})}_{{\varvec{K}}{\varvec{L}}\boldsymbol{ }0}]}$$

### Step 3: Automatic detection of osteophytes

MediAI-OA was trained to automatically classify the presence of osteophytes and mark the location of osteophytes in each of the four ROIs by using a NASNet model, which is an image classification model based on convolutional neural network [25].

### Step 4: Automatic evaluation of KL grade

In addition, MediAI-OA was developed to automatically classify KL grade using NASNet model. NASNet was used because the NASNet model showed higher sensitivity and specificity for discriminating each KL grade than the EfficientNet and ResNet models. Due to the fact that it is not very clinically important to distinguish between KL grades 0 and 1, this model was designed to Groups 0 and 1 together.

### *Step5: I*ntegration and visualization of knee OA features

**Fig. 6:**
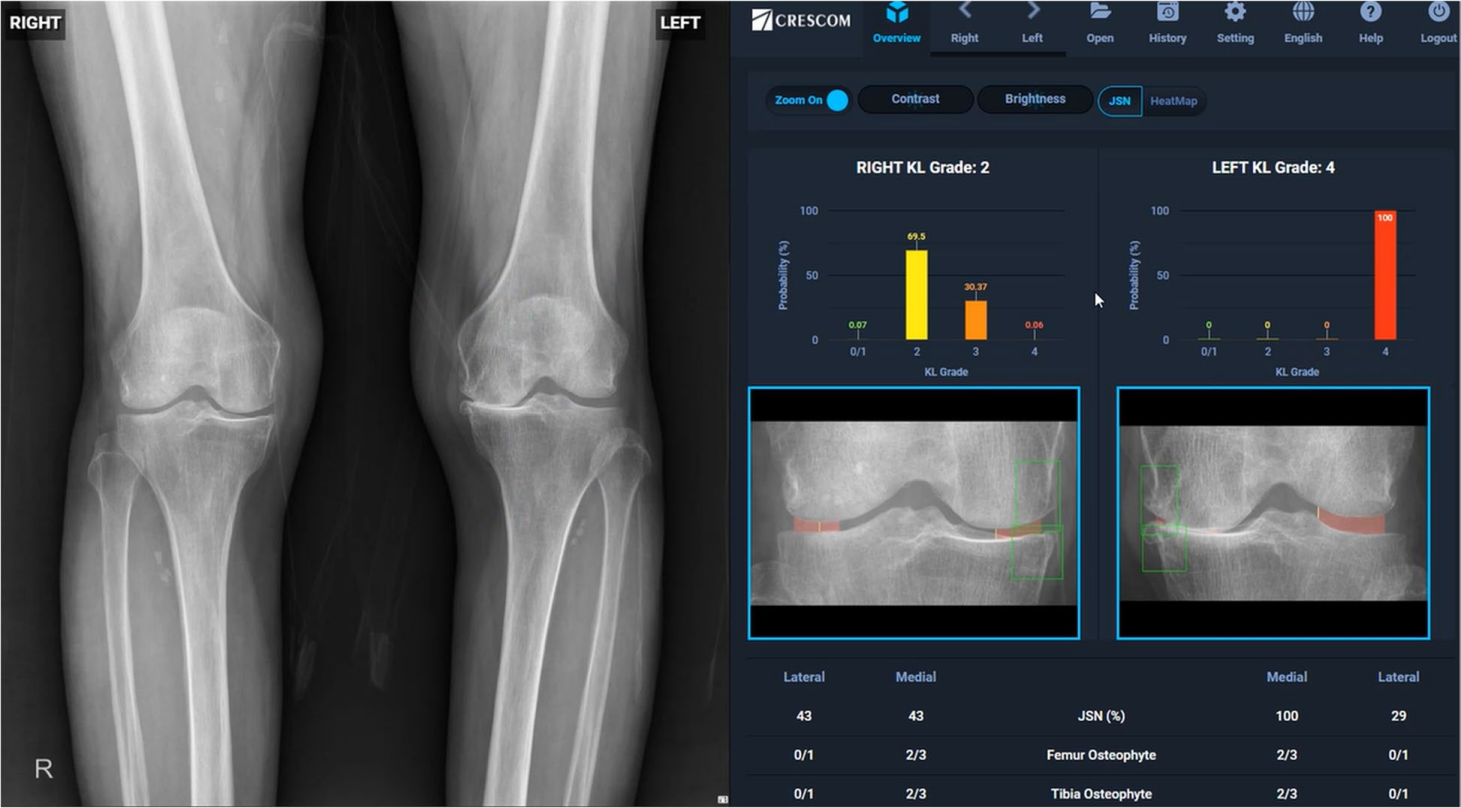
Example of result presented by MediAI-OA

After automatically acquiring the results of the JSN rate, presence of osteophytes and KL grade, this AI integrated and visualized the three results of OA features together (Fig. 6).

### Performance assessment

To assess the performance of the AI model, the model was tested by using the test dataset, and the results were compared to the ground truth. The accuracy of mJSW quantification, JSN rate quantification and osteophyte detection were determined. The precision, recall, F1 score and accuracy of automatic KL grading were evaluated [26, 27]. In addition, precision, recall and F1 score for OA diagnosis were obtained. KL grades 0 and 1 were classified as normal knees, and KL grades 2, 3 and 4 were classified as OA [7, 18, 28].

The accuracy of mJSW quantification and JSN rate quantification were calculated using mean squared error (MSE) as follows:

$${{\varvec{M}}{\varvec{S}}{\varvec{E}}}_{{\varvec{m}}{\varvec{J}}{\varvec{S}}{\varvec{W}}}=\boldsymbol{ }\frac{1}{{\varvec{n}}}\sum^{{\varvec{n}}}{({{\varvec{m}}{\varvec{J}}{\varvec{S}}{\varvec{W}}}_{{\varvec{p}}{\varvec{r}}{\varvec{e}}{\varvec{d}}{\varvec{i}}{\varvec{c}}{\varvec{t}}{\varvec{i}}{\varvec{o}}{\varvec{n}}}-{{\varvec{m}}{\varvec{J}}{\varvec{S}}{\varvec{W}}}_{{\varvec{g}}{\varvec{r}}{\varvec{o}}{\varvec{u}}{\varvec{n}}{\varvec{d}}\boldsymbol{ }{\varvec{t}}{\varvec{r}}{\varvec{u}}{\varvec{t}}{\varvec{h}}})}^{2}$$

$${{\varvec{M}}{\varvec{S}}{\varvec{E}}}_{{\varvec{J}}{\varvec{S}}{\varvec{N}}\_{\varvec{r}}{\varvec{a}}{\varvec{t}}{\varvec{e}}}=\boldsymbol{ }\frac{1}{{\varvec{n}}}\sum^{{\varvec{n}}}{({{\varvec{J}}{\varvec{S}}{\varvec{N}}\boldsymbol{ }{\varvec{r}}{\varvec{a}}{\varvec{t}}{\varvec{e}}}_{{\varvec{p}}{\varvec{r}}{\varvec{e}}{\varvec{d}}{\varvec{i}}{\varvec{c}}{\varvec{t}}{\varvec{i}}{\varvec{o}}{\varvec{n}}}-{{\varvec{J}}{\varvec{S}}{\varvec{N}}\boldsymbol{ }{\varvec{r}}{\varvec{a}}{\varvec{t}}{\varvec{e}}}_{{\varvec{g}}{\varvec{r}}{\varvec{o}}{\varvec{u}}{\varvec{n}}{\varvec{d}}\boldsymbol{ }{\varvec{t}}{\varvec{r}}{\varvec{u}}{\varvec{t}}{\varvec{h}}})}^{2}$$

The osteophyte detection accuracy of each of the four ROIs was calculated by using the following equation, and the average accuracy was also calculated:

$${Accuracy}_{osteophyt{e}_{i}}= \frac{{True Positive}_{i}+{True Negative}_{i}}{{True Positive}_{i}+{True Negative}_{i}+{False Positive}_{i}+{False Negative}_{i}}$$

$$(i=MF, LF, MT, LT)$$

The accuracy of automatic KL grading was calculated by dividing the number of knees classified as true positive KL grade by the total number of knees. In addition, Cohen’s kappa coefficient (к) was calculated to evaluate the agreement between MediAI-OA and our experienced physicians who established the ground truth. A к value of 0–0.4 is considered fair, 0.4–0.6 is considered moderate, 0.6–0.8 is considered substantial and 0.8–1 is considered almost perfect agreement.

## Results

### Automatic segmentation of joint space & quantification

The MSE for mJSW quantification and JSN rate quantification of the MediAI-OA model were 0.000082 and 0.067, respectively.

### Automatic detection of osteophytes

The average osteophyte detection accuracy of this model was 0.84. Among the four ROIs, the MT region showed the highest accuracy of 0.93, and the LF region showed the lowest accuracy of 0.69 (Table 1).

**Table 1:** Accuracy of osteophyte detection in 4 zones

Zone	MF	LF	MT	LT	Average
Accuracy	0.84	0.69	0.93	0.90	0.84

*MF* Medial distal femur, *LF* Lateral distal femur, *MT* Medial proximal tibia, *LT* Lateral proximal tibia

### Automatic KL grading

**Fig. 7:**
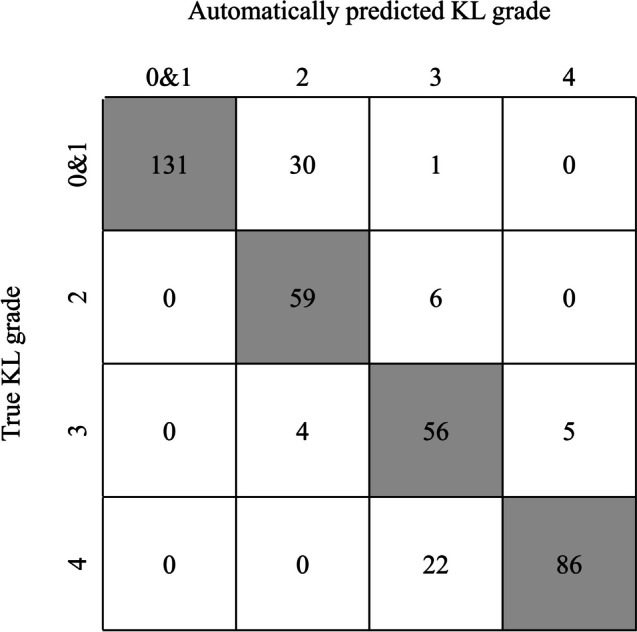
Confusion matrix of the MediAI-OA performance test. The true KL grade is the ground truth result determined by the experienced physician. The automatically predicted KL grade is the result of KL grade classification via MediAI-OA. The gray columns show the number of test data for which the result of MediAI-OA and the ground truth matched

The precision, recall and F1 score for each KL grade are shown in Table 2. The confusion matrix of our model is presented in Fig. 7, and the accuracy of KL grading was 0.83. The к value between the AI model and ground truth was 0.768, which demonstrated substantial consistency.

**Table 2:** Performance of MediAI-OA for KL grading

KL Grade	Number of test set	Precision	Recall	F1-score
KL 0 & 1	162	1.00	0.80	0.89
KL 2	65	0.63	0.91	0.74
KL 3	65	0.77	0.74	0.76
KL 4	108	0.89	0.94	0.91

*KL* Kellgren-Lawrence

### Diagnosis of osteoarthritis

The F1 scores for the diagnosis of no OA and OA were 0.89 and 0.94, respectively (Table 3). The overall OA diagnosis accuracy of this model was 0.92.

**Table 3:** Performance of MediAI-OA for OA diagnosis

	Number of test set	Precision	Recall	F1-score
No OA (KL 0 + 1)	162	1.00	0.80	0.89
OA (KL 2 + 3 + 4)	238	0.88	1.00	0.94

*OA* Osteoarthritis, *KL* Kellgren-Lawrence

## Discussion

In this study, we developed a novel DL software known as MediAI-OA that automatically quantifies the JSN rate, detects osteophytes and classifies KL grade. MediAI-OA showed high accuracy in measuring JSN rate, detecting osteophytes, determining KL grade and diagnosing OA.

The differences between MediAI-OA and other previously introduced DL softwares that automatically classify KL grades are as follows. 1) To reduce the effect of individual knee size, a technique to quantify mJSW relative to tibial width was introduced. 2) This technique was developed to obtain the JSN rate compared to normal knees. 3) The JSN rate, the presence of osteophytes and KL grade, which are characteristics of OA, are integrated and presented together. Therefore, MediAI-OA is advantageous for future software modifications, and clinicians can use this software to help in determining OA classification. In previous study, there was also a model showing OA characteristics along with KL grade [29]. In this model, JSW was expressed in ‘mm’ units, and JSN was divided into four levels. The biggest difference from previous software model is that MediAI-OA quantitatively analyzes and presents the JSN rate as a percentage compared to normal. This allows physicians to more accurately track the progression of joint space narrowing. Additionally, this study analyzes and presents the exact performance of each KL grade.

The average accuracy of the automatic KL classification of MediAI-OA was 0.83, which is superior to the reported accuracy values of 0.66 to 0.72 in previous DL software studies [11–13, 15, 16, 30]. In addition, when MediAI-OA was used to classify KL Grade < 2 and ≥ 2, it showed a near perfect accuracy of 0.92. In order to reduce uncertainty and increase reliability of the DL model, this model not only provides KL grade, but also analyzes and provides information such as the grade of osteophytes and the JSN rate compared to normal population.

In the results of this study, the F1 scores of KL grades 2 and 3 were relatively low compared to other grades. This result is thought to be due to the ambiguity of the definition of joint space narrowing. In this study, the ground truth was defined as JSN of < 50% for “possible joint space narrowing” corresponding to KL grade 2 and > 50% for “definite joint space narrowing” corresponding to KL grade 3. However, the cutoff value of JSN of "possible" and "definite” joint space narrowing that central leaders of OAI think may not be exactly 50%. Therefore, further research is needed to determine the quantitative rates of JSN of “possible” and “definite” conditions that experienced clinicians have assessed.

The osteophyte detection accuracy of our model was relatively low in LF compared to other regions. This is likely due to the fact that the bony morphology of the lateral distal femur is not smooth due to the popliteal groove, and the contour varies depending on the angle of the X-ray beam, after which double contour occurs [31]. This may also be the reason that the lateral border of the lateral femoral condyle is a site where the morphological difference between the AP view and the Rosenberg view is large.

This study had several limitations. First, our model only detected the presence of osteophytes in the 4 ROIs but did not discriminate the severity of osteophytes. In addition, osteophytes can also occur in areas other than the four ROIs that we set. However, due to the fact that the ROIs that we set are the most common osteophyte-occurring regions and most important regions for K-L grading, they will not have a significant impact on the results. Second, MediAI-OA cannot evaluate subchondral sclerosis or bony abnormalities. Due to the fact that these features are also included in KL grading, it will be necessary to develop future models to evaluate these OA features together. Third, this model can only detect tibiofemoral joint OA on knee AP or Rosenberg radiographs. Additional research will be needed to develop a model that can also automatically determine OA of the patellofermoral joint through knee lateral and skyline radiographs. Fourth, this software does not distinguish between KL grade 0 and 1. However, in clinical practice, the distinction between KL grade 0 and 1 is not meaningful because it does not have a major impact on determining the diagnosis and treatment policy. Fifth, in this model, three characteristics are automatically analyzed and each result is just visualized together. In the next version, we are developing a model that integrates the results of the three OA features and performs final KL grading.

Through this DL software, more reliable diagnoses and OA grades can be provided to patients even in clinics without musculoskeletal radiologists or orthopedic specialists. Additionally, the joint space area and osteophyte locations are displayed visually and intuitively, and the JSN rate is presented as a percentage, so that patients could more easily understand their knee condition. Even when following a patient, it can provide more accurate information about the progression of OA on radiograph than past radiographs. This model can be used to reduce the time radiologists spend interpreting plain knee radiographs.

## Conclusions

The novel DL software MediAI-OA demonstrated satisfactory performance comparable to that of experienced orthopedic surgeons and radiologists for analyzing features of knee OA, KL grading and OA diagnosis. Therefore, reliable KL grading can be performed by using MediAI-OA, and the burden of the radiologist can be reduced.

## Data Availability

The datasets used for training and validation of the software in this study are OAI data and are publicly available from https://nda.nih.gov/oai/.

## References

[1] Hunter DJ, March L, Chew M (2020). Osteoarthritis in 2020 and beyond: a lancet commission. Lancet.

[2] Hunter DJ, Bierma-Zeinstra S (2019). Osteoarthritis. Lancet.

[3] Hong JW, Noh JH, Kim D-J (2020). The prevalence of and demographic factors associated with radiographic knee osteoarthritis in Korean adults aged ≥ 50 years: The 2010–2013 Korea National Health and Nutrition Examination Survey. PLoS ONE.

[4] Cui A, Li H, Wang D, Zhong J, Chen Y, Lu H (2020). Global, regional prevalence, incidence and risk factors of knee osteoarthritis in population-based studies. EClinicalMedicine.

[5] Kontis V, Bennett JE, Mathers CD, Li G, Foreman K, Ezzati M (2017). Future life expectancy in 35 industrialised countries: projections with a Bayesian model ensemble. Lancet.

[6] Kellgren JH, Lawrence JS (1957). Radiological assessment of osteo-arthrosis. Ann Rheum Dis.

[7] Kohn MD, Sassoon AA, Fernando ND (2016). Classifications in brief: Kellgren-Lawrence classification of osteoarthritis. Clin Orthop Relat Res.

[8] Riddle DL, Jiranek WA, Hull JR (2013). Validity and reliability of radiographic knee osteoarthritis measures by arthroplasty surgeons. Orthopedics.

[9] Wright RW, Group M (2014). Osteoarthritis classification scales: interobserver reliability and arthroscopic correlation. J Bone Joint Surg Am.

[10] Köse Ö, Acar B, Çay F, Yilmaz B, Güler F, Yüksel HY (2018). Inter- and intraobserver reliabilities of four different radiographic grading scales of osteoarthritis of the knee joint. J Knee Surg.

[11] Swiecicki A, Li N, O'Donnell J, Said N, Yang J, Mather RC (2021). Deep learning-based algorithm for assessment of knee osteoarthritis severity in radiographs matches performance of radiologists. Comput Biol Med.

[12] Tiulpin A, Saarakkala S (2020). Automatic grading of individual knee osteoarthritis features in plain radiographs using deep convolutional neural networks. Diagnostics (Basel).

[13] Thomas KA, Kidziński Ł, Halilaj E, Fleming SL, Venkataraman GR, Oei EHG (2020). Automated Classification of radiographic knee osteoarthritis severity using deep neural networks. Radiol Artif Intell.

[14] Tiulpin A, Thevenot J, Rahtu E, Lehenkari P, Saarakkala S (2018). Automatic knee osteoarthritis diagnosis from plain radiographs: a deep learning-based approach. Sci Rep.

[15] Olsson S, Akbarian E, Lind A, Razavian AS, Gordon M (2021). Automating classification of osteoarthritis according to Kellgren-Lawrence in the knee using deep learning in an unfiltered adult population. BMC Musculoskelet Disord.

[16] Helwan A, Azar D, Abdellatef H (2022). An update on the knee osteoarthritis severity grading using wide residual learning. J Xray Sci Technol.

[17] Lester G (2008). Clinical research in OA–the NIH osteoarthritis Initiative. J Musculoskelet Neuronal Interact..

[18] Schiphof D, Boers M, Bierma-Zeinstra SM (2008). Differences in descriptions of Kellgren and Lawrence grades of knee osteoarthritis. Ann Rheum Dis.

[19] Guermazi A, Hayashi D, Roemer F, Felson DT, Wang K, Lynch J (2015). Severe radiographic knee osteoarthritis – does Kellgren and Lawrence grade 4 represent end stage disease? – the MOST study. Osteoarthritis Cartilage.

[20] Yamashita R, Nishio M, Do RKG, Togashi K (2018). Convolutional neural networks: an overview and application in radiology. Insights Imaging.

[21] Tang Z, Gao Y, Karlinsky L, Sattigeri P, Feris R, Metaxas D. OnlineAugment: online data augmentation with less domain knowledge. In: Vedaldi A, Bischof H, Brox T, Frahm JM, editors. Computer vision – ECCV 2020. ECCV 2020. Springer, Cham; 2020. p. 313–329. (Lecture Notes in Computer Science; vol 12352).

[22] Wang J, Sun K, Cheng T, Jiang B, Deng C, Zhao Y (2021). Deep high-resolution representation learning for visual recognition. IEEE Trans Pattern Anal Mach Intell.

[23] Lin T-Y, Goyal P, Girshick R, He K, Dollar P. Focal loss for dense object detection. IEEE Trans Pattern Anal Mach Intell. 2020;42(2):318–27. 10.1109/TPAMI.2018.2858826 30040631

[24] Paixao T, DiFranco MD, Ljuhar R, Ljuhar D, Goetz C, Bertalan Z (2020). A novel quantitative metric for joint space width: data from the Osteoarthritis Initiative (OAI). Osteoarthritis Cartilage.

[25] Zoph B, Vasudevan V, Shlens J, Le QV. Learning transferable architectures for scalable image recognition. Proc IEEE Comput Soc Conf Comput Vis Pattern Recognit. 2018; 8697–8710.

[26] Hicks SA, Strümke I, Thambawita V, Hammou M, Riegler MA, Halvorsen P (2022). On evaluation metrics for medical applications of artificial intelligence. Sci Rep.

[27] Na S, Sung YS, Ko Y, Shin Y, Lee J, Ha J (2022). Development and validation of an ensemble artificial intelligence model for comprehensive imaging quality check to classify body parts and contrast enhancement. BMC Med Imaging.

[28] Norman B, Pedoia V, Noworolski A, Link TM, Majumdar S (2019). Applying Densely connected convolutional neural networks for staging osteoarthritis severity from plain radiographs. J Digit Imaging.

[29] Nehrer S, Ljuhar R, Steindl P, Simon R, Maurer D, Ljuhar D (2021). Automated knee osteoarthritis assessment increases physicians' agreement rate and accuracy: data from the osteoarthritis initiative. Cartilage.

[30] Chen P, Gao L, Shi X, Allen K, Yang L (2019). Fully automatic knee osteoarthritis severity grading using deep neural networks with a novel ordinal loss. Comput Med Imaging Graph.

[31] Guermazi A, Hunter DJ, Li L, Benichou O, Eckstein F, Kwoh CK (2012). Different thresholds for detecting osteophytes and joint space narrowing exist between the site investigators and the centralized reader in a multicenter knee osteoarthritis study–data from the Osteoarthritis Initiative. Skeletal Radiol.

